# Correlation between exercise, personal income level and health-related quality of life in patients with newly diagnosed stable angina

**DOI:** 10.1186/s40779-019-0226-5

**Published:** 2019-11-25

**Authors:** Yi Wang, Lei Huang, Lai-Xin Zhou

**Affiliations:** 1 Department of Cardiology, the Second Affiliated Hospital of Army Medical University, Chongqing, 400037 China; 2 Division of Medical Affairs, the Second Affiliated Hospital of Army Medical University, Chongqing, 400037 China; 3 The First Affiliated Hospital of Army Medical University, Chongqing, 400038 China

**Keywords:** Coronary outpatients, Exercise, Health-related quality of life, Monthly income

## Abstract

**Background:**

Stable angina is one of the most common clinical types of coronary artery disease (CAD) and associated with poor health-related quality of life (HRQL). However, few studies have evaluated the risk factors associated with HRQL in patients with newly diagnosed stable angina.

**Methods:**

A cross-sectional survey was conducted with 342 consecutive outpatients with newly diagnosed stable angina from October 2017 to January 2018 at the Second Affiliated Hospital of Army Medical University, Chongqing, China. Eight dimensions of HRQL were evaluated via the 36-item Short-Form Health Survey, including physical functioning, role limitation due to physical problems, bodily pain, general health, vitality, social functioning, role limitation due to emotional problems, and mental health. Physical and mental component summaries were calculated. Multiple stepwise regression was performed to determine the factors associated with HRQL.

**Results:**

Patients who were older, were females, did not exercise, had lower educational levels, had lower monthly incomes, had smoking/drinking habits, and had diabetes, hypertension, or hyperlipemia showed lower physical HRQL scores, while those who were older with lower educational levels and lower monthly incomes showed lower mental HRQL scores. The results of the multiple stepwise regression analyses showed that physical and mental HRQL were positively correlated with exercise and monthly income and negatively associated with age. Patients with monthly income ≥5000 Yuan showed higher HRQL scores than those with monthly income < 5000 Yuan. Sleep quality and drinking were negatively associated with physical, but not mental HRQL.

**Conclusions:**

Our findings indicated that exercise and personal income level, both modifiable factors, were positively associated with physical and mental HRQL. These findings could have implications for clinical suggestions and strategies to improve HRQL in patients with stable angina.

## Background

Coronary artery disease (CAD) is the most common clinical type of cardiovascular disease, and the leading cause of morbidity and disability worldwide [1, 2]. Because of considerable advances in the treatment and management of CAD in recent decades, the number of CAD patients in low- and middle-income countries continues to increase [3, 4]. Moreover, some research has shown that patients with CAD exhibited poor health-related quality of life (HRQL) [5, 6], which is an important indicator in evaluating patient-centered health outcomes, the impact of disease burden, and the effectiveness of treatment interventions. Nevertheless, evidence regarding factors associated with HRQL in CAD patients using outpatient services is lacking, particularly with respect to common factors in daily life.

HRQL is a self-reported outcome of individual health and well-being, including subjective symptoms, general health, functional status, and satisfaction with life [7, 8]. Based on the notion that health is more than the absence of disease, HRQL evaluation has become a major public health topic over recent decades. According to the latest research, age, chronic disease, health services, medical insurance, education, economic status, and physical exercise are the main factors influencing HRQL in the general population; in particular, age and disease are independent risk factors [9, 10]. However, the factors that influence HRQL in CAD outpatients and the consistency of these factors between physical and mental HRQL remain unclear.

Therefore, the current study aimed to assess the associations between these factors and HRQL and explore predictive relationships in CAD outpatients. The findings could provide valuable evidence for medical decision-making in the development of strategies to improve HRQL in CAD outpatients.

## Methods

### Ethics statement

The study protocol was approved by the ethics committee on human experimentation at the Hospital of Army Medical University, Chongqing, China (NO: 2018-YD078–01) and conformed to the standards established by the Declaration of Helsinki. All participants read and signed the consent form.

### Study design and sample

We conducted a cross-sectional survey between October 2017 and January 2018, with 342 consecutive outpatients with stable angina from the outpatient Department of Cardiology at the Second Affiliated Hospital of Army Medical University, Chongqing, China. According to a previous definition [11, 12], patients with chronic stable angina and/or ischemia, as demonstrated via stress tests, exhibited stenosis of any diameter greater than 70% in at least one epicardial vessel. The exclusion criteria included lesions in the left main coronary artery; multivessel disease; acute coronary syndrome; and previous myocardial infarction, percutaneous coronary intervention, or coronary artery bypass grafting. Participants with serious comorbid illnesses, such as immunological diseases, cognitive impairment, renal failure requiring hemodialysis and cancer, and those who could not understand the questionnaire sufficiently were also excluded from the study.

The sample size was predetermined, and the statistical power was calculated by using the PASS software, version 11 (NCSS, LLC, Kaysville, UT, USA), which suggested that 342 consecutive outpatients would provide more than 80% power for the present study.

### Participants’ basic characteristics

The following general characteristics were recorded and categorized: age (≤45, 46–60, or > 60 years), gender (male or female), body mass index (BMI; normal: 18.5–23.9 kg/m^2^; overweight: 24.0–27.9 kg/m^2^; obese: ≥28.0 kg/m^2^), educational level (bachelor’s degree or lower, or master’s degree or higher), monthly income (< 5000 or ≥ 5000 Yuan), marital status (married, unmarried, or divorced), smoking (yes or no), drinking (yes or no), exercise (never, once per week, or twice per week or more), work/rest cycle (regular or irregular), sleep quality (good or poor), diabetes (fasting blood glucose≥126 mg/ml or 7 mmol/L, or use of a specific pharmacological treatment), hypertension (systolic blood pressure of ≥140 mmHg, diastolic blood pressure of ≥90 mmHg, or treatment for hypertension), hyperlipemia (total cholesterol ≥240 mg/dl or 6.2 mmol/L, or use of lipid-lowering drugs). We defined poor sleep quality as follows: insomnia, < 5 h sleep per night or use of sleeping medication. Responses regarding chronic disease (excluding diabetes, hypertension and hyperlipemia) were limited to yes or no.

### HRQL measurement

We distributed the scales to the selected population in the outpatient department, asked them to fill it out under the guidance of the research assistant and then analyzed the data after recycling. The 36-Item Short Form health survey (SF-36; Mandarin version), which has been identified as a reliable and valid measurement tool for HRQL (Cronbach’s alpha ranging from 0.75 to 0.90), was used to assess physical and mental HRQL [13, 14]. The SF-36 includes 36 items and assesses eight dimensions of HRQL including physical functioning (PF), role-physical (RP), bodily pain (BP), general health (GH), vitality (VT), social functioning (SF), role limitation due to emotional problems (RE), and mental health (MH). The physical component summary (PCS) is calculated as the sum of PF, RP, BP, and GH scores, and the mental component summary (MCS) is calculated as the sum of VT, SF, RE, and MH scores. Higher scores, which indicate better health, are calculated and transformed into a value ranging from 0 to 100 [15, 16].

### Statistical analysis

We used SPSS 18.0 (SPSS Inc., Chicago, IL, USA) to perform two-sample *t* tests, one-way ANOVAs, or Kruskal-Wallis *H* tests as appropriate, and multivariate stepwise regression was performed to evaluate the factors influencing HRQL. In addition, we performed *t* tests and ANOVAs to evaluate the distribution of HRQL for the categorical variables. We also performed Pearson’s correlation analysis to assess correlations between HRQL scores and continuous variables. Collinearity between two variables was considered to exist when the correlation coefficient for the relationship between the variables was *P* > 0.50. No collinear variables were identified, and multivariate stepwise regression was conducted using HRQL clusters as dependent variables and basic characteristics as independent variables. Data are expressed as the means ± standard deviations (SD), and the significance level was set at *P* < 0.05.

## Results

### Basic characteristics

Data were obtained from 342 outpatients with stable angina. The participants’ basic characteristics are shown in Table 1. The age of the participants ranged from 30 to 65 years. There were 209 (61.1%) males and 133 (38.9%) females. Of the 342 participants, 173 (50.6%) participants had a normal BMI, 138 (40.4%) participants were overweight, and 31 (9.1%) participants were obese; 41 (12.0%) participants had a master or higher degree, and 301 (88.0%) participants had a bachelor’s or lower degree. The monthly income of 290 (84.8%) participants was lower than 5000 Yuan; 52 (15.2%) participants earned more than 5000 Yuan every month. Regarding participant marital status, 274 (80.1%) participants were married, and 68 (19.9%) participants were single or divorced. 222 (64.9%) participants were smokers, and 212 (62.0%) participants were drinkers. With respect to their exercising habits, 132 (38.6%) participants never exercised, 93 (27.2%) participants exercised once per week, and 117 (34.2%) participants exercised twice or more per week. 121 (35.4%) participants had a regular work/rest cycle, whereas the rest had an irregular cycle, and 108 (31.6%) participants had good sleep quality, while the rest did not. Forty (11.7%) participants had diabetes, 110 (32.2%) participants had hypertension, and 55 (16.1%) participants had hyperlipemia.

**Table 1:** PF, RP, BP, GH, and PCS scores based on the characteristics of patients with newly diagnosed stable angina

Item	*n* (%)	PF (score)	RP (score)	BP (score)	GH (score)	PCS (score)
Gender						
Male	209 (61.1)	47.1 ± 36.7	50.0 ± 33.8	62.8 ± 25.3	49.1 ± 18.7	52.3 ± 18.3
Female	133 (38.9)	36.7 ± 27.2^*^	42.3 ± 33.6^*^	59.0 ± 24.5	48.8 ± 16.8	46.7 ± 17.9^*^
Age (years)						
≤ 45	84 (24.6)	59.2 ± 29.5	60.1 ± 32.6	68.2 ± 22.9	54.4 ± 20.9	60.5 ± 17.0
46–60	129 (37.7)	42.7 ± 30.2^*^	49.1 ± 33.7^*^	61.3 ± 25.5	50.4 ± 17.1	50.9 ± 18.5^*^
> 60	129 (37.7)	32.9 ± 29.8^*#^	36.5 ± 31.7^*#^	57.0 ± 25.1^*^	44.0 ± 15.3^*#^	42.6 ± 15.4^*#^
BMI (kg/m^2^)						
≤ 23.9 (Normal)	173 (50.6)	42.0 ± 30.3	45.1 ± 32.8	60.0 ± 26.8	47.7 ± 18.7	48.7 ± 19.0
24.0–27.9 (Overweight)	138 (40.4)	44.4 ± 29.2	47.2 ± 35.1	65.3 ± 23.1	50.3 ± 17.8	51.8 ± 17.9
≥ 28.0 (Obese)	31 (9.1)	42.7 ± 30.1	57.3 ± 33.3	51.7 ± 20.1^#^	50.1 ± 13.6	50.5 ± 16.0
Educational level						
Bachelor’s degree or lower	301 (88.0)	41.4 ± 28.8	44.4 ± 34.0	60.0 ± 25.1	48.1 ± 17.3	48.5 ± 17.5
Master’s degree or higher	41 (12.0)	54.9 ± 34.2^*^	66.0 ± 34.6^*^	71.2 ± 22.6^*^	55.3 ± 21.4^*^	61.9 ± 17.1^*^
Monthly income (Yuan)						
< 5000	290 (84.8)	40.5 ± 29.1	43.6 ± 33.7	60.1 ± 25.5	48.5 ± 17.8	48.2 ± 18.1
≥ 5000	52 (15.2)	57.5 ± 29.9^*^	66.3 ± 27.7^*^	68.3 ± 21.5^*^	51.9 ± 18.6	61.0 ± 15.5^*^
Marital status						
Married	274 (80.1)	43.1 ± 29.2	49.0 ± 33.5	61.4 ± 25.5	47.9 ± 18.1	50.4 ± 18.2
Unmarried/Divorced	68 (19.9)	42.9 ± 32.2	38.9 ± 33.4^*^	61.3 ± 23.2	53.3 ± 16.8^*^	49.1 ± 18.8
Smoking						
No	120 (35.1)	49.5 ± 28.1	55.4 ± 30.9	62.4 ± 22.2	50.6 ± 17.6	54.5 ± 16.4
Yes	222 (64.9)	39.6 ± 30.1^*^	42.5 ± 34.5^*^	60.8 ± 26.5	48.1 ± 18.1	47.7 ± 18.9^*^
Drinking						
No	130 (38.0)	49.8 ± 27.9	55.2 ± 32.6	62.5 ± 23.5	52.1 ± 17.6	54.9 ± 17.0
Yes	212 (62.0)	38.9 ± 30.2^*^	42.0 ± 33.7^*^	60.6 ± 26.0	47.1 ± 17.9^*^	47.2 ± 18.5^*^
Exercise						
Never	132 (38.6)	35.6 ± 29.2	34.3 ± 30.6	58.4 ± 26.4	44.9 ± 16.3	43.6 ± 16.8
Once per week	93 (27.2)	49.1 ± 29.2^*^	59.4 ± 32.6^*^	63.0 ± 23.1	51.8 ± 18.6^*^	55.8 ± 17.4^*^
Twice per week or more	117 (34.2)	45.5 ± 31.2^*^	51.6 ± 33.7^*^	63.4 ± 24.8	51.3 ± 18.4^*^	52.9 ± 18.5^*^
Work/Rest cycle						
Regular	121 (35.4)	41.7 ± 28.0	46.0 ± 34.0	62.0 ± 25.4	49.4 ± 17.9	49.8 ± 18.1
Irregular	221 (64.6)	43.8 ± 30.7	47.6 ± 33.8	61.0 ± 24.9	48.8 ± 18.0	50.3 ± 18.4
Sleep quality						
Good	108 (31.6)	49.2 ± 30.9	54.0 ± 35.2	67.1 ± 22.9	52.4 ± 20.6	55.7 ± 18.7
Poor	234 (68.4)	40.2 ± 28.9^*^	43.8 ± 32.8^*^	58.7 ± 25.6^*^	47.4 ± 16.4^*^	47.5 ± 17.6^*^
Diabetes						
No	302 (88.3)	44.1 ± 30.7	48.3 ± 34.3	61.8 ± 24.8	49.5 ± 17.9	50.9 ± 18.4
Yes	40 (11.7)	35.5 ± 20.7^*^	37.5 ± 29.0^*^	58.1 ± 26.9	44.8 ± 17.6	44.0 ± 16.3^*^
Hypertension						
No	232 (67.8)	44.2 ± 30.9	47.0 ± 34.7	64.5 ± 24.3	51.0 ± 18.9	51.7 ± 18.6
Yes	110 (32.2)	40.6 ± 27.3	47.2 ± 32.1	54.6 ± 25.4^*^	44.8 ± 14.9^*^	46.8 ± 17.4^*^
Hyperlipemia						
No	287 (83.9)	42.8 ± 30.3	48.1 ± 34.5	62.5 ± 24.9	50.1 ± 18.3	50.9 ± 18.7
Yes	55 (16.1)	44.6 ± 27.1	41.5 ± 29.8	55.2 ± 25.3^*^	42.9 ± 14.3^*^	46.1 ± 15.6^*^

^*^P < 0.05 vs the first row in the same category; ^#^*P* < 0.05 vs the second row in the same category; *PF* Physical functioning, *RP* Role-physical, *BP* Bodily pain, *GH* General health, *PCS* Physical component summary, *SD* Standard deviation, *BMI* Body mass index

### Physical and mental HRQL

The results regarding physical HRQL are shown in Table 1. There were gender differences in PF, RP, and PCS scores, and men displayed higher scores relative to those of women. PF, RP, BP, GH, and PCS scores decreased significantly with age, particularly in those aged > 60 years. Outpatients with a master’s degree or higher exhibited higher PF, RP, BP, GH, and PCS scores relative to those observed in participants with a bachelor’s degree or lower. Regarding monthly income, participants who earned ≥5000 Yuan showed higher PF, RP, BP, and PCS scores relative to those observed for participants who earned < 5000 Yuan. Married participants’ RP scores were higher, and their GH scores were lower, relative to those observed in unmarried participants. Drinkers’ PF, RP, GH and PCS scores were lower relative to those of non-drinkers, and smokers’ PF, RP, and PCS scores were lower relative to those of nonsmokers. Furthermore, participants who exercised regularly showed higher PF, RP, GH, and PCS relative to those observed in participants who never exercised, but these scores were higher in those who exercised once per week higher than those who exercised twice per week. Participants who reported poor sleep showed lower PF, RP, BP, GH, and PCS scores relative to those observed in participants who reported good sleep. Participants with diabetes exhibited lower PF, RP and PCS scores relative to those observed in participants without diabetes, and participants with hypertension or hyperlipemia displayed lower BP, GH, and PCS relative to those observed in participants without these illnesses. No significant differences were observed according to BMI or work/rest cycle.

Mental HRQL results are shown in Table 2. There was a gender difference in VT scores, with men displaying higher scores relative to those of women. VT, SF, RE, and MCS scores decreased significantly with age, particularly in participants aged > 60 years. Regarding educational level, participants with a master’s degree or higher showed higher VT, MH, and MCS scores relative to those observed in participants with a bachelor’s degree or lower. VT, SF, MH, and MCS scores observed for participants who earned ≥5000 Yuan were higher relative to those observed in participants who earned < 5000 Yuan. The smokers’ VT and MH scores were lower relative to those observed in nonsmokers, and the drinkers’ VT scores were lower relative to those observed in non-drinkers. Furthermore, relative to those observed in participants who never exercised, participants who exercised once per week displayed higher VT and MH scores, and participants who exercised at least twice per week exhibited higher VT and RE scores. Participants who reported poor sleep showed lower VT sores relative to those observed in participants who reported good sleep. Participants with hypertension displayed lower SF scores relative to those observed in participants without these illnesses. No significant differences were observed according to BMI, marital status, work/rest cycle, diabetes, or hyperlipemia.

**Table 2:** VT, SF, RE, MH, and MCS scores according to the characteristics of patients with newly diagnosed stable angina

Item	*n* (%)	VT (score)	SF (score)	RE (score)	MH (score)	MCS (score)
Gender						
Male	209 (61.1)	48.3 ± 19.9	66.5 ± 18.8	71.5 ± 27.1	59.9 ± 17.6	61.6 ± 13.7
Female	133 (38.9)	43.5 ± 20.1^*^	64.7 ± 21.7	67.7 ± 31.3	59.9 ± 18.7	58.9 ± 14.1
Age (years)						
≤ 45	84 (24.6)	51.9 ± 20.5	69.5 ± 17.4	75.5 ± 25.7	58.8 ± 18.4	63.9 ± 13.4
46–60	129 (37.7)	47.9 ± 20.5	67.0 ± 20.5	70.9 ± 29.3	61.1 ± 18.1	61.7 ± 14.1
> 60	129 (37.7)	41.4 ± 18.3^*#^	62.2 ± 20.5^*^	65.6 ± 29.7^*^	59.3 ± 17.7	57.1 ± 13.3^*#^
BMI (kg/m^2^)						
≤ 23.9 (Normal)	173 (50.6)	45.1 ± 20.8	65.1 ± 20.5	67.8 ± 31.1	58.9 ± 18.6	59.2 ± 14.7
24.0–27.9 (Overweight)	138 (40.4)	48.0 ± 19.9	68.5 ± 18.8	73.5 ± 26.1	62.5 ± 17.4	63.1 ± 12.2
≥ 28.0 (Obese)	31 (9.1)	46.9 ± 16.5	57.7 ± 19.8^#^	66.9 ± 26.4	53.6 ± 15.5^#^	56.3 ± 14.2^#^
Educational level						
Bachelor’s degree or lower	301 (88.0)	45.6 ± 19.8	65.1 ± 20.2	69.5 ± 28.9	59.2 ± 18.2	59.9 ± 13.8
Master’s degree or higher	41 (12.0)	52.8 ± 21.4^*^	70.7 ± 17.9	73.4 ± 28.2	64.7 ± 16.3^*^	65.4 ± 13.2^*^
Monthly income (Yuan)						
< 5000	290 (84.8)	44.8 ± 20.1	64.8 ± 20.5	69.3 ± 29.5	59.1 ± 18.6	59.5 ± 13.9
≥ 5000	52 (15.2)	55.6 ± 17.4^*^	71.4 ± 15.5^*^	73.7 ± 24.5	64.4 ± 13.7^*^	66.3 ± 12.1^*^
Marital status						
Married	274 (80.1)	46.8 ± 19.2	66.1 ± 19.6	70.2 ± 28.3	59.4 ± 17.7	60.6 ± 13.9
Unmarried/Divorced	68 (19.9)	45.2 ± 23.3	64.7 ± 21.6	69.1 ± 31.2	61.7 ± 19.1	60.2 ± 13.8
Smoking						
No	120 (35.1)	51.4 ± 16.8	63.9 ± 17.2	71.4 ± 25.0	57.0 ± 16.9	60.9 ± 12.4
Yes	222 (64.9)	43.8 ± 21.2^*^	66.8 ± 21.3	69.3 ± 30.7	61.5 ± 18.4^*^	60.3 ± 14.5
Drinking						
No	130 (38.0)	51.7 ± 18.1	64.5 ± 17.9	73.3 ± 25.2	57.8 ± 16.7	61.8 ± 12.8
Yes	212 (62.0)	43.3 ± 20.6^*^	66.6 ± 21.2	68.0 ± 30.7	61.2 ± 18.7	59.7 ± 14.4
Exercise						
Never	132 (38.6)	41.4 ± 21.5	66.7 ± 23.2	64.5 ± 32.0	61.4 ± 18.9	58.5 ± 15.5
Once per week	93 (27.2)	51.2 ± 16.6^*^	66.7 ± 16.5	72.2 ± 27.2	55.3 ± 15.2^*^	61.2 ± 12.3
Twice per week or more	117 (34.2)	48.4 ± 19.8^*^	64.6 ± 18.6	74.4 ± 25.3^*^	61.8 ± 18.5	62.3 ± 12.9
Work/rest cycle						
Regular	121 (35.4)	46.0 ± 20.4	66.0 ± 19.3	69.8 ± 29.8	60.9 ± 17.7	60.7 ± 13.6
Irregular	221 (64.6)	46.7 ± 19.9	65.7 ± 20.4	70.1 ± 28.3	59.3 ± 18.2	60.5 ± 14.0
Sleep quality						
Good	108 (31.6)	50.8 ± 18.7	68.0 ± 17.7	70.5 ± 29.0	59.5 ± 15.8	62.2 ± 13.3
Poor	234 (68.4)	44.4 ± 20.4^*^	64.8 ± 20.9	69.8 ± 28.8	60.0 ± 19.0	59.7 ± 14.1
Diabetes						
No	302 (88.3)	46.9 ± 20.1	66.5 ± 19.4	70.8 ± 28.5	59.9 ± 17.7	61.0 ± 13.5
Yes	40 (11.7)	43.3 ± 19.6	60.6 ± 23.5	64.2 ± 30.6	59.9 ± 20.5	57.0 ± 15.9
Hypertension						
No	232 (67.8)	46.6 ± 20.5	68.2 ± 19.3	69.7 ± 29.4	60.5 ± 17.7	61.2 ± 13.7
Yes	110 (32.2)	46.1 ± 19.2	60.7 ± 20.6^*^	70.7 ± 27.6	58.5 ± 18.7	59.0 ± 14.2
Hyperlipemia						
No	287 (83.9)	46.9 ± 20.7	66.0 ± 20.5	69.2 ± 29.2	59.7 ± 18.2	60.4 ± 14.2
Yes	55 (16.1)	44.3 ± 16.8	64.9 ± 17.0	74.2 ± 26.3	61.0 ± 17.2	61.1 ± 12.1

^*^P < 0.05 vs the first row in the same category; ^#^*P* < 0.05 vs the second row in the same category; *VT* Vitality, *SF* Social functioning, *RE* Role limitation due to emotional problems, *MH* Mental health, *MCS* Mental component summary, *SD* Standard deviation, *BMI* Body mass index

### Factors associated with physical and mental HRQL

Multivariate stepwise regression was performed to identify the potential factors that might be independently associated with HRQL. Among the eight SF-36 dimensions, the scores for PF, RP, BP and GH were summarized into the PCS, which represented the global physical HRQL score. The scores for VT, SF, RE and MH were summarized into the MCS, which represented the global mental HRQL. PCS and MCS were used as the dependent variables, and the risk factors in Tables 1 and 2 were used as independent variables. The results of the stepwise multiple regression analysis showed that the PCS scores were negatively associated with age, sleep quality, and drinking but positively associated with exercise and monthly income (Table 3). Moreover, the MCS scores were also positively associated with exercise and monthly income, but they were only negatively associated with age (Table 4).

**Table 3:** Stepwise regression predicting PCS scores

Variables	Physical Component Summary (PCS)				
	Step 1 (β)	Step 2 (β)	Step 3 (β)	Step 4 (β)	Step 5 (β)
Age	−0.524^**^	−0.518^**^	−0.479^**^	− 0.456^**^	− 0.430^**^
Exercise		4.569^**^	4.342^**^	4.045^**^	4.133^**^
Monthly income			7.379^**^	7.844^**^	7.417^**^
Sleep quality				−5.701^**^	−5.725^**^
Drinking					−4.678^**^

^*^P < 0.05, ^**^P < 0.01

**Table 4:** Stepwise regression predicting the MCS scores

Variables	Mental Component Summary (MCS)		
	Step 1 (β)	Step 2 (β)	Step 3 (β)
Age	−0.224^**^	−0.199^**^	− 0.198^**^
Monthly income		4.840^*^	4.534^*^
Exercise			3.556^*^

^*^P < 0.05, ^**^P < 0.01

## Discussion

Improvement in HRQL for patients with CAD is always valuable and beneficial. It is worthwhile to understand which factors can affect the outcomes of HRQL and how these factors can be used to optimize CAD interventions. Our present study involving outpatients with stable angina demonstrated that physical HRQL was positively associated with exercise and monthly income and negatively associated with age, sleep quality, and drinking, while mental HRQL was positively associated with monthly income and exercise and negatively associated with age. Our findings suggested that patients with stable angina who perform moderate exercise and have a higher income level could benefit more in HRQL, although aging cannot be postponed.

The disease burden of CAD is usually evaluated by measuring HRQL, and the results of a longitudinal cohort study showed that approximately 26% of CAD patients experienced a significant reduction in HRQL over a 5-year period [17, 18]. Moreover, the framework developed by the International Classification of Functioning, Disability and Health demonstrated that reductions in HRQL in CAD patients involve not only physical symptoms with activity limitations but also social support, participation, and personal perception [19, 20]. Angina is the initial clinical manifestation in 25 to 50% of all CAD patients [21, 22] and has been associated with poor HRQL and depressive symptoms; individuals with angina exhibit higher scores for pain or worry relative to those observed in individuals without angina [23–26]. Furthermore, reduced HRQL has been associated with poor prognosis in CAD patients [27, 28]. Therefore, exploration of risk factors affecting HRQL scores in patients with newly diagnosed stable angina is of great importance. However, to our knowledge, the disease burden and risk factors for newly diagnosed stable angina, as evaluated via HRQL, have not been examined comprehensively.

The current results showed that physical and mental HRQL were positively associated with exercise and monthly income and negatively associated with age. Among these factors, monthly income was the strongest factor associated with not only physical HRQL but also mental HRQL, and patients with a monthly income ≥5000 Yuan showed higher HRQL scores than those of patients with a monthly income < 5000 Yuan. Indeed, personal or family income level has been previously associated with reduced HRQL in some chronic medical conditions and cardiovascular diseases [29–32]. Patients with lower income may have a limited ability to obtain effective treatments, which may improve their clinical outcomes. Such conditions may ultimately result in poor HRQL. Moreover, we also found that patients who exercised at least once a week had higher physical and mental HRQL scores than patients who never exercised, and the stepwise regression also showed that exercise was positively associated with physical and mental HRQL. These findings are consistent with those of a previous study indicating that poor HRQL was associated with greater fatigue and decreased exercise capacity, independent of mental distress and CAD severity, in CAD patients undergoing rehabilitation [33]. Another study revealed that both age and household income were associated with HRQL; however, gender, perceived social support, history of angina, and dyslipidemia were identified as risk factors [34, 35]. This partial inconsistency could be attributed to differences between the participants. Previous studies reported that comorbid conditions (i.e., heart failure and peripheral artery disease), the frequency with which patients visited family physicians, and educational level were significant predictors of HRQL in CAD patients [5]. Factors such as heart failure, peripheral artery disease, and the frequency with which participants visited family physicians were not included in the current study; however, comorbid conditions (i.e., diabetes, hypertension, and hyperlipemia) and educational level were not identified as risk factors for poor HRQL in patients with stable angina. Furthermore, sleep quality and drinking were negatively associated with physical, but not mental HRQL, which is consistent with previous results [36].

Our present findings made us think about the possible interventions for the improvement of HRQL in this type of patient in practice. Although age, which was unmodifiable, was also a significant factor associated with poor HRQL, other factors identified in our present study could be modified to improve the HRQL as well as the patient-centered clinical outcomes. Doing exercises seemed to be the most feasible and economical method to improve both physical and mental HRQL. Additionally, we can also improve the quality of physical life by improving sleep quality and increasing average income. Moreover, the government should be under an obligation to increase average income in the general population.

### Limitations

This study was subject to some limitations. For example, the cross-sectional study design did not allow inference of causal relationships between variables. Furthermore, the use of self-reported assessments could have led to bias in the data analysis, although the reliability and validity of the SF-36 have been evaluated extensively. Moreover, because the study sample included only one type of participant, caution should be exercised in generalizing the results to the general population.

## Conclusions

This study showed that physical health status was associated with age, sleep quality, exercise habits, and monthly income, while mental health status was associated with age, exercise, and monthly income in outpatients with newly diagnosed stable angina. Higher monthly income was associated with higher physical and mental HRQL scores, while regular exercise was related to higher PCS scores. These findings are important in planning strategies to improve physical and mental health in cardiovascular medicine outpatients. Future research should involve a large survey with random samples of outpatients with newly diagnosed stable angina worldwide.

## Data Availability

The datasets generated and/or analyzed in this study are available at the Second Affiliated Hospital of Army Medical University, Chongqing, China.

## Abbreviations

BMI: Body mass index
BP: Bodily pain
CAD: Coronary artery disease
GH: General health
HRQL: Health-related quality of life
MCS: Mental component summary
MH: Mental health
PCS: Physical component summary
PF: Physical functioning
RE: Role limitation due to emotional problems
RP: Role-physical
SD: Standard deviation
SF: Social functioning
SF-36: 36-Item Short-Form Health Survey
VT: Vitality
